# Comparative prognosis of pediatric lymphoblastic lymphoma: insights from Chinese and international cohorts

**DOI:** 10.3389/fonc.2025.1626143

**Published:** 2025-12-01

**Authors:** Luyao Zhang, Jianwen Zhou, Yongyan He, Tianhui Chen, Wei Liu

**Affiliations:** 1 Henan Provincial Clinical Research Center for Pediatric Diseases, Children's Hospital Affiliated to Zhengzhou University, Henan Children's Hospital, Zhengzhou Children's Hospital, Zhengzhou, China; 2 College of Public Health, Zhengzhou University, Zhengzhou, Henan, China; 3 Department of Hematology Oncology, Children’s Hospital Affiliated to Zhengzhou University, Zhengzhou University, Zhengzhou, China; 4 Department of Cancer Prevention, Zhejiang Cancer Hospital, Hangzhou, China; 5 Hangzhou Institute of Medicine (HIM), Chinese Academy of Sciences, Hangzhou, China

**Keywords:** T-lymphoblastic lymphoma, B-lymphoblastic lymphoma, pediatric, lymphoblastic lymphoma, prognosis

## Abstract

**Objective:**

The aim of this research was to characterize the clinical features and histological subtypes of pediatric lymphoblastic lymphoma (LBL) and to assess the clinical prognostic factors for 178 pediatric patients. Data from two independent cohorts were used: the China-LBL cohort and the United States Surveillance, Epidemiology, and End Results (SEER)-LBL cohort.

**Methods:**

A retrospective analysis was conducted using SEER data and a single-center cohort of pediatric patients from China. Survival analysis and prognostic factor evaluations were performed to identify patterns and discrepancies between the two cohorts. Patients diagnosed with LBL from both the China-LBL and SEER-LBL cohorts were included. Statistical analyses involved the chi-square test, Kaplan-Meier method, and multivariate Weibull regression for survival analysis.

**Results:**

The study identified 77 patients in the China-LBL cohort and 101 patients in the SEER cohort. In the China-LBL cohort, 60 (77.9%) were T-LBL and 17 (22.1%) were B-LBL. In the SEER-LBL cohort, 65 (64.4%) were T-LBL and 36 (35.6%) were B-LBL. The highest proportion of patients was observed in stage IV in both cohorts (China-LBL: 80.5%; SEER-LBL: 48.5%). The overall survival between B-LBL and T-LBL patients was not significantly different in either cohort (SEER: P = 0.79; China: P = 0.14). Furthermore, patients treated during 2017–2019 had significantly better overall survival compared to those treated between 2020–2023 in both the entire LBL cohort (P <.001) and the T-LBL subgroup (P = 0.003) of the China cohort. Multivariate analysis did not identify any independent prognostic factors in either the SEER or China cohort. The overall survival of LBL patients in the China cohort showed statistically significant differences between the periods of 2017–2019 and 2020-2023, across gender, age, histology, and primary site groups.

**Conclusion:**

Pediatric lymphoblastic lymphoma (LBL) is predominantly T-cell subtype (China: 77.9%, SEER: 64.4%) and frequently diagnosed at stage IV. Survival did not differ between B- and T-LBL, but China cohort patients treated in 2017–2019 had better outcomes than those in 2020–2023 (P<0.01), possibly linked to COVID-19 disruptions. No independent prognostic factors were identified, warranting larger studies integrating treatment details to refine risk stratification.

## 1 Introduction

Lymphoblastic lymphoma (LBL) are progenitor malignancies with 70%-80% originating from T-lymphoblasts and 20%-25% from B-lymphoblasts (1). According to the World Health Organization (WHO) classification of hematolymphoid tumors, both acute lymphoblastic leukemia (ALL) and LBL are considered to be part of the same disease spectrum, differentiated primarily by the threshold of 25% lymphoblasts in the bone marrow (BM) (2). Despite this classification, the question of whether pediatric LBL and ALL are biologically identical or distinct entities remains a subject of ongoing debate in the field. Moreover, increasing evidence from molecular and proteomic studies suggests significant differences between T-ALL and T-LBL (3–6), prompting a reevaluation of their distinct pathophysiology. Genetic driver mutations and differentially expressed genes (DEGs) suggest that T-LBL and T-ALL may arise through distinct pathogenic mechanisms and rely on disparate molecular determinants (7, 8). However, despite this recognition, the current understanding of these differences remains fragmented and lacks a systematic explanation.

Both T-LBL and T-ALL originate from T-lineage cells, and their immunophenotype is defined by the specific stage of differentiation at which arrest occurs. Briefly, multipotent hematopoietic progenitors (MPPs) and common lymphoid progenitors (CLPs) emerge from the bone marrow and migrate to the thymus. Here, they undergo a stepwise differentiation process, progressing through the stages of early T-cell precursors, double-negative (DN), double-positive (DP), and finally CD4+ or CD8+ single-positive (SP) T cells (9, 10). Although research has identified differences in immunophenotypes between T-LBL and T-ALL, the molecular mechanisms underlying LBL are still not fully understood, which complicates the development of targeted therapeutic strategies. While treatment regimens for ALL have been widely applied in LBL, leading to improvements in outcomes, there is significant variation in event-free survival (EFS) rates, with reports ranging from 75% to 90% (11–13). This variability can be attributed to differences in clinical features, genetic mutations, and treatment protocols. Additionally, while advancements in chemotherapy have contributed to improved survival, there remain notable regional and ethnic disparities in clinical presentations and prognostic outcomes (14, 15). These disparities underscore the necessity of a more nuanced understanding of how geographic, socioeconomic, and genetic factors influence the clinical behavior and prognosis of pediatric LBL.

The T-cell and B-cell subtypes of LBL exhibit distinct biological profiles, clinical manifestations, and responses to therapy (16). Despite significant progress in understanding the disease, the variation in treatment outcomes across different regions and populations suggests that further investigation into these disparities is critical. However, large-scale, multicenter, and multinational studies comparing the outcomes of pediatric LBL patients from diverse cohorts remain relatively scarce. As such, retrospective studies examining the clinical features, prognostic factors, and survival outcomes of pediatric LBL are urgently needed, particularly in regions with varying healthcare infrastructures.

This study aims to conduct a comparative analysis of the clinical features, histologic subtypes, and survival outcomes of pediatric LBL patients from two distinct cohorts: the China-LBL cohort and the SEER-LBL cohort. By analyzing these cohorts, we seek to identify potential prognostic factors and assess any differences in treatment response, thereby contributing valuable insights to the understanding of LBL outcomes and informing future therapeutic strategies.

## 2 Materials and methods

### 2.1 Data source

In this retrospective study, we used data from the Chinese Single-Center cohort and the SEER 8 registries cohort of the National Cancer Institute. The Chinese Single-Center cohort includes data of patients diagnosed with LBL who underwent pathological detection between April 3, 2017, and September 26, 2023, at a provincial-level tertiary children’s hospital in China. Ethical approval for this study was obtained from the institutional review board of the hospital (Ethical approval number: 2024-102-001).

The SEER Program registries routinely collect data on patient demographics, primary tumor site, and stage at diagnosis, as well as follow-up information for vital status in the United States. Data on patients diagnosed with LBL between January 1, 2000, and December 31, 2009, were obtained from the SEER 18 Registries using the SEER*Stat software (version 8.4.4). Patients with a first primary diagnosis of LBL (aged 0–18 years) were identified using the International Classification of Diseases for Oncology, 3rd edition (ICD-O-3) codes (morphology codes: 9729/3 for T-LL, 9728/3 for B-LL). Additional selection criteria included: (1) First malignant primary diagnosis: Yes; (2) Known survival time; (3) Age 0–18 years; (4) Known lymphoma Ann Arbor stage. Clinical information and data used in this study were all downloaded from a publicly available database, and therefore, ethical approval and informed consent were waived.

The study periods for the two cohorts differ due to the nature of their data sources. The SEER cohort includes patients from 2000 to 2009, as this represents the period of consistent and reliable data coverage for the specific ICD-O-3 codes (9728/3 for B-LBL and 9729/3 for T-LBL) within the utilized SEER dataset. The China-LBL cohort, in contrast, comprises consecutive patients from a single center treated between 2017 and 2023.

### 2.2 Procedures and outcomes

This study is a retrospective cohort study that included clinical data of children with LBL who were treated for the first time in the hematology department of a provincial-level tertiary children’s hospital in China from April 2017 to November 2021. Demographic, clinical, and outcome data were extracted from the institution’s electronic medical records by uniformly trained personnel. Standardized data collection templates were used to minimize selection bias. Inclusion criteria: Diagnosed with B-LBL or T-LBL according to the WHO 2008 pathological classification criteria. Age <18 years. Exclusion criteria: Recurrent B-LBL or T-LBL. Treatment at an external hospital for more than one week before admission. Diagnosis of this disease as the child’s second tumor.

#### 2.2.1 Data collection

Clinical data, including age at onset, gender, disease duration, initial symptoms, site of invasion, clinical staging, risk grouping, treatment plan, and treatment response, were collected through the hospital’s electronic medical record system.

#### 2.2.2 Diagnostic criteria for histological types

Tissue pathology, immunophenotyping, cytogenetics, and molecular biology tests were performed on biopsies from the affected tissue. The diagnosis could only be confirmed after consultation with pathology experts from at least two tertiary hospitals (one expert from each hospital), provided that their results were consistent. The diagnosis and classification followed the WHO 2008 pathological diagnostic criteria.

#### 2.2.3 Clinical staging

Before chemotherapy, patients underwent whole-body imaging, bone marrow biopsy (from at least two locations, including the iliac bone), and lumbar puncture to assess tumor invasion. Clinical staging was based on the St. Jude staging system.

#### 2.2.4 Treatment plan

Lymphoblastic lymphoma (LBL) shares many biological characteristics and prognostic features with acute T lymphoblastic leukemia (T-ALL). The World Health Organization (WHO) classifies both conditions together as precursor T-cell lymphoma/leukemia (T-LBL/ALL).

Chemotherapy regimens, adapted based on risk stratification, have led to a 5-year event-free survival (EFS) rate of 75%-90% in international multicenter studies (16–19). Among these regimens, the Berlin-Frankfurt-Munster 90-LBL (BFM-90-LBL) protocol has been one of the most effective treatments for pediatric and adolescent LBL. Since January 2003, most children’s hospitals in northern China have adopted a modified version of the BFM-90-LBL protocol, known as the BCH-2003-LBL regimen, which has resulted in a 3-year EFS rate of 85.4% (20). In the cohort of Chinese patients included in this study, the treatment regimen was further modified with the BCH-2017-LBL protocol, based on the BFM-95-LBL protocol. This modification omitted cranial radiation therapy (CRT), even for patients with central nervous system (CNS) involvement. All patients in this study received chemotherapy based on the BCH-2017-LBL regimen, which included induction remission therapy, early intensified therapy, consolidation therapy, delayed intensified therapy, and maintenance therapy. CNS prevention and treatment were administered based on the patient’s CNS status.

#### 2.2.5 Treatment status of children in different risk groups

Stratified chemotherapy was applied according to the risk groups. There was no delayed intensified treatment for stage I and II patients, except for stage II patients with early spontaneous tumor dissolution or large tumor masses. Stage III and IV patients received chemotherapy according to the medium-risk group regimen. Whether patients entered the high-risk group was determined by evaluating their treatment response and the presence of prognostic genes after treatment. For example, patients with the presence of the *MLL/AF4* fusion gene, other *MLL* rearrangements, the *BCR::ABL1* fusion gene, or Ph-like ALL-related gene abnormalities (including but not limited to *IKZF1, CRLF2*, and *JAK2*) were classified into the high-risk group.

#### 2.2.6 Clinicopathological variables

The clinicopathological variables analyzed in this study included: age, stage, radiotherapy, chemotherapy, and survival in months. Age was categorized as <5 years, 5–10 years, and >10 years. Staging information for the SEER cohort was based on the Lymphoma Ann Arbor Staging system. Radiotherapy was classified as yes, no, or unknown. Chemotherapy was classified as yes, no, or unknown according to the SEER classification. Data on race and ethnicity were not collected in this study.

#### 2.2.7 Primary outcome

The primary outcome of the study in both cohorts was overall survival (OS), defined as the length of time from the date of first diagnosis until the date of death from any cause, or the last follow-up if the patient was still alive at the end of the study period.

### 2.3 Statistical analysis

The baseline categorical characteristics between B-LBL and T-LBL histology groups were compared with χ² test or Fisher’s exact test. The Kaplan-Meier method was used to construct survival curves, and log-rank test for trend was used for determining the statistical significance of the difference in Kaplan-Meier survival. Multivariable Weibull regression analyses was used for multivariable survival analyses. All variables (sex, age, stage, primary site, radiotherapy, and chemotherapy) were included in multivariable Weibull regression analyses. Hazard ratios (HR) are reported with 95% CIs. All tests were two-sided with statistical significance set at p less than 0.05.

We selected the Weibull regression model for survival analysis due to its flexibility and suitability for handling censored data, particularly in situations where the follow-up period is limited or sample sizes are smaller. In this study, the Chinese cohort had a shorter follow-up period and a smaller number of patients, which made traditional survival models like Cox proportional hazards regression less ideal for capturing the complex relationships between variables. The Weibull model, being more adaptable to different types of survival data distributions, provides a robust alternative for survival analysis in smaller cohorts with limited follow-up. Additionally, the model allows for the inclusion of both continuous and categorical variables, making it a comprehensive tool for analyzing prognostic factors in the pediatric LBL population.

All analyses were performed using R statistical software (version 4.4.4).

## 3 Results

A total of 178 patients were included in the study: 101 patients from the SEER database and 77 patients from the Chinese single-center cohort. The median follow-up time was 180 months (IQR 156–204) for the SEER cohort and 8 months (IQR 3–24) for the Chinese cohort (**Table 1**). The proportion of patients with stage I disease was much lower in the Chinese cohort (1 patient, 1.3%) compared to the SEER cohort (19 patients, 18.8%). Treatment methods also differed between the two cohorts. In the SEER cohort, 21 patients (20.8%) underwent radiotherapy, while no patients in the Chinese cohort received radiotherapy.

**Table 1:** Clinicopathological characteristics of patients from the SEER database and China single center data.

Variables	SEER cohort (n=101)	China cohort (n=77)
Sex		
Male	56 (55.4%)	57 (74.0%)
Female	45 (44.6%)	20 (26.0%)
Age, years		
< 5 years	25 (24.8%)	23 (30.0%)
5–10 years	36 (35.6%)	36 (46.8%)
> 10 years	40 (39.6%)	18 (23.4%)
Year of diagnosis		
2000-2004	40 (39.6%)	NA
2005-2009	61 (60.4%)	NA
2017-2019	NA	28
2020-2023	NA	49
Histology		
T-LBL	65 (64.4%)	60 (77.9%)
B-LBL	36 (35.6%)	17 (22.1%)
Primary site		
Nodal NHL	80 (79.2%)	62 (80.5%)
Extra-nodal NHL	21 (20.8%)	15 (19.5%)
Stage		
I	19 (18.8%)	1 (1.3%)
II	17 (16.8%)	NA
III	16 (15.8%)	14 (18.2%)
IV	49 (48.5%)	62 (80.5%)
Radiotherapy		
Yes	21 (20.8%)	NA
None/Unknown	80 (79.2%)	77 (100.0%)
Chemotherapy		
Yes	100 (99.0%)	77 (100.0%)
None/Unknown	1(1.00%)	NA
Median follow-up time, months (IQR)	180 (156-204)	8 (3-24)

SEER, Surveillance, Epidemiology, and End Results; T-LBL, T-cell lymphoblastic lymphoma; B-LBL, B-cell lymphoblastic lymphoma.

In terms of age distribution, more T-LBL patients in the Chinese cohort were in the 5–10 years and older age groups (p<0.001; **Table 2**). However, in the SEER cohort, the majority of B-LBL patients were in the >10 years age group. In the SEER cohort, more T-LBL patients underwent radiotherapy compared to B-LBL patients (p=0.002; **Table 2**). B-LBL patients were more prevalent in both stage I and stage IV in the SEER cohort. In contrast, in the Chinese cohort, the majority of T-LBL patients were concentrated in stages III and IV.

**Table 2:** Baseline characteristics of eligible patients, stratified by histology.

Variables	SEER cohort database			China cohort		
	B-LBL (n=36)	T-LBL (n=65)	P value	LBL (n=17)	T-LBL (n=60)	P value
Sex						
Male	17 (47.2%)	37 (56.9%)	0.467	12 (70.6%)	45 (75.0%)	0.958
Female	19 (52.8%)	28 (43.1%)		5 (29.4%)	15 (25.0%)	
Age, years						
< 5 years	9 (25.0%)	16 (24.6%)	0.930	12 (70.6%)	11 (18.3%)	<.001
5–10 years	12 (33.3%)	24 (36.9%)		4 (23.5%)	32 (53.3%)	
> 10 years	15 (41.7%)	25 (38.5%)		1 (5.9%)	17 (28.3%)	
Year of diagnosis						
2000-2004	14 (38.9%)	26 (40.0%)	1.000	NA	NA	
2005-2009	22 (61.1%)	39 (60.0%)		NA	NA	
2017-2019	NA	NA		5 (29.4%)	23 (38.3%)	0.697
2020-2023	NA	NA		12 (70.6%)	37 (61.7%)	
Primary site						
Nodal NHL	20 (55.6%)	60 (92.3%)	<.001	12 (70.6%)	50 (83.3%)	0.410
Extra-nodal NHL	16 (44.4%)	5 (7.7%)		5 (29.4%)	10 (16.7%)	
Stage						
I	11 (30.6%)	8 (12.3%)	0.002	1 (5.9%)	0	<.001
II	2 (5.6%)	13 (20.0%)		4 (23.5%)	0	
III	1 (2.8%)	15 (23.1%)		3 (17.6%)	11 (18.3%)	
IV	20 (55.6%)	29 (44.6%)		9 (52.9%)	49 (81.7%)	
Radiotherapy						
Yes	1 (2.8%)	20 (30.8%)	0.002	0	0	1.000
None/Unknown	35 (97.2%)	45 (69.2%)		17 (100.0%)	60 (100.0%)	
Chemotherapy						
Yes	36 (100.0%)	64 (98.5%)	1.000	17 (100.0%)	60 (100.0%)	1.000
None/Unknown	0	1 (1.5%)		0	0	
Overall survival						
Alive	29 (80.6%)	54 (83.1%)	0.964	17 (100.0%)	51 (85.0%)	0.194
Dead	7 (19.4%)	11 (16.9%)		0	9 (15.0%)	

SEER, Surveillance, Epidemiology, and End Results; T-LBL, T-cell lymphoblastic lymphoma; B-LBL, B-cell lymphoblastic lymphoma.

In multivariate analysis, no independent prognostic factors were identified in either the SEER or Chinese cohort. However, statistical differences in prognosis were observed between patients from different time periods in the Chinese cohort (HR = 35.07, 95%CI: 5.1–241.26, P<0.001; **Table 3**), with a relatively wide confidence interval. Similar findings were observed in the Chinese T-LBL cohort (HR = 18.5, 95%CI: 2.74–124.95, P = 0.003; **Table 4**).

**Table 3:** Adjusted clinical prognostic factors for children lymphoblastic lymphoma.

Variables	SEER cohort			China cohort		
	Multivariate			Multivariate		
	Hazard ratio	95%CI	P value	Hazard ratio	95%CI	P value
Age, years						
< 5 years	Reference			Reference		
5–10 years	0.74	0.17-3.16	0.679	0.18	0.02-1.94	0.158
> 10 years	1.94	0.53-7.13	0.316	0.36	0.04-3.30	0.364
Gender						
Female	Reference			Reference		
Male	0.99	0.35-2.78	0.986	11.88	0.74-191.13	0.081
Primary site						
Nodal NHL	Reference			Reference		
Extra-nodal NHL	0.58	0.15-2.30	0.443	NA	NA	1.000
Stage						
I	Reference					
II	0.35	0.06-2.01	0.239			
III	0.58	0.11-2.97	0.517	Reference		
IV	0.50	0.14-1.76	0.282	0.33	0.06-1.73	0.189
Year of diagnose						
2000-2004	Reference					
2005-2009	1.94	0.65-5.74	0.232			
2017-2019				Reference		
2020-2023				35.07	5.1-241.26	<.001
Radiotherapy						
Yes	Reference					
None/Unknown	0.83	0.22-3.05	0.776			

SEER, Surveillance, Epidemiology, and End Results; T-LBL, T-cell lymphoblastic lymphoma; B-LBL, B-cell lymphoblastic lymphoma; CI, Confidence interval.

**Table 4:** Adjusted clinical prognostic factors for children lymphoblastic lymphoma by histology type.

Variables	T-LBL (SEER cohort)			T-LBL (China cohort)		
	Multivariate			Multivariate		
	Hazard ratio	95%CI	P-value	Hazard ratio	95%CI	P-value
Age, years						
< 5 years	Reference			Reference		
5–10 years	1.47	0.23-9.33	0.685	0.24	0.02-2.38	0.222
> 10 years	2.04	0.34-12.43	0.423	0.34	0.04-2.97	0.331
Gender						
Female	Reference			Reference		
Male	1.5	0.32-7.11	0.609	6.32	0.44-90.5	0.175
Primary site						
Nodal NHL	Reference			Reference		
Extra-nodal NHL	NA	NA	1.000	NA	NA	1.000
Stage						
I	Reference					
II	0.38	0.04-3.83	0.413			
III	0.29	0.03-3.19	0.310	Reference		
IV	0.38	0.05-2.95	0.352	0.26	0.05-1.4	0.118
Year of diagnose						
2000-2004	Reference					
2005-2009	2.19	0.53-9.04	0.277			
2017-2019				Reference		
2020-2023				18.5	2.74-124.95	0.003
Radiotherapy						
Yes	Reference					
None/Unknown	0.93	0.23-3.78	0.915			

SEER, Surveillance, Epidemiology, and End Results; T-LBL, T-cell lymphoblastic lymphoma; B-LBL, B-cell lymphoblastic lymphoma; CI, Confidence interval.

**Figure 1:**
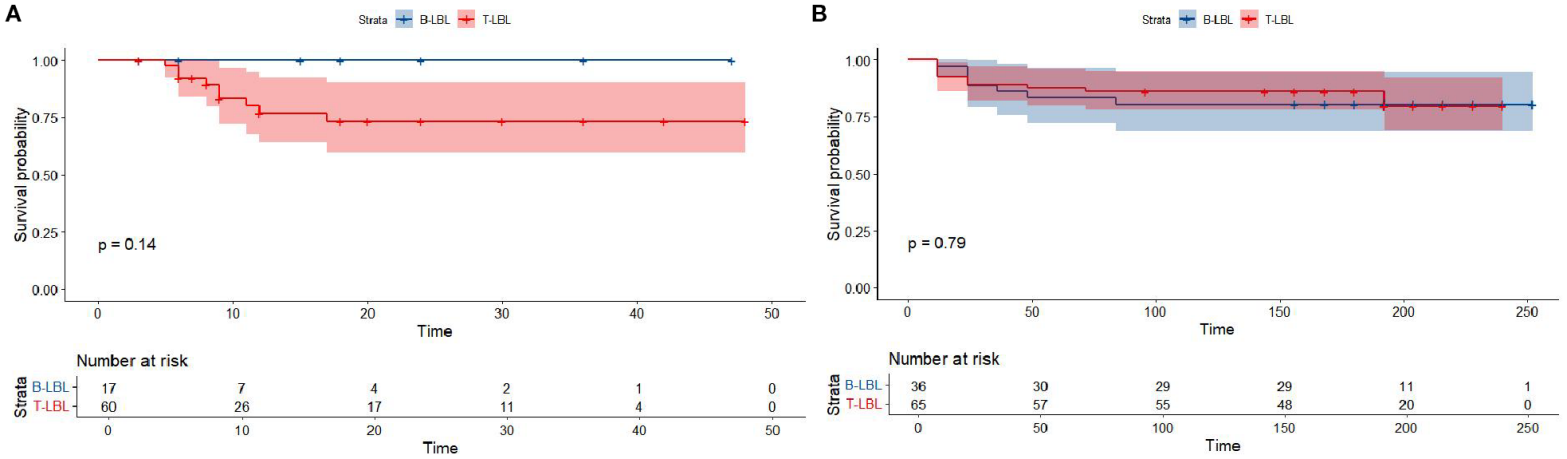
Overall survival in participants with Lymphoblastic Lymphoma by histology type from the SEER cohort **(A)** and Chinese cohort **(B)**.

**Figure 2:**
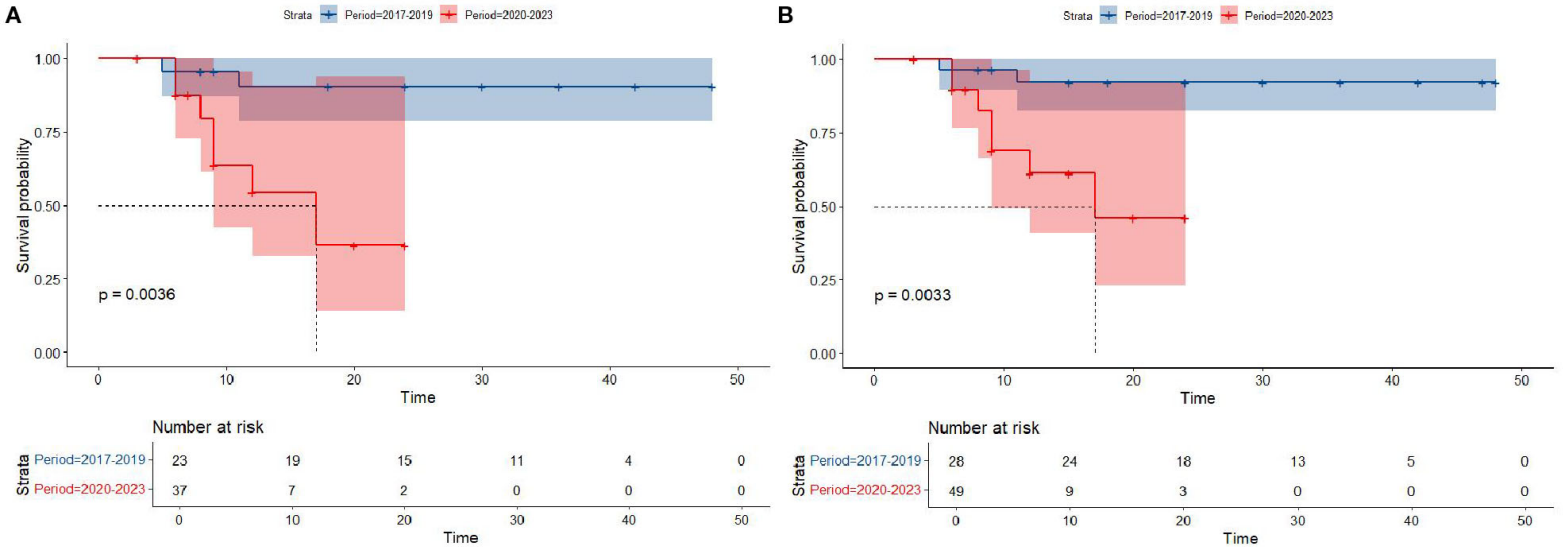
Overall survival in participants with Lymphoblastic Lymphoma from the Chinese cohort by period year (T-LBL: **(A)**; All-LBL: **(B)**).

**Figure 3:**
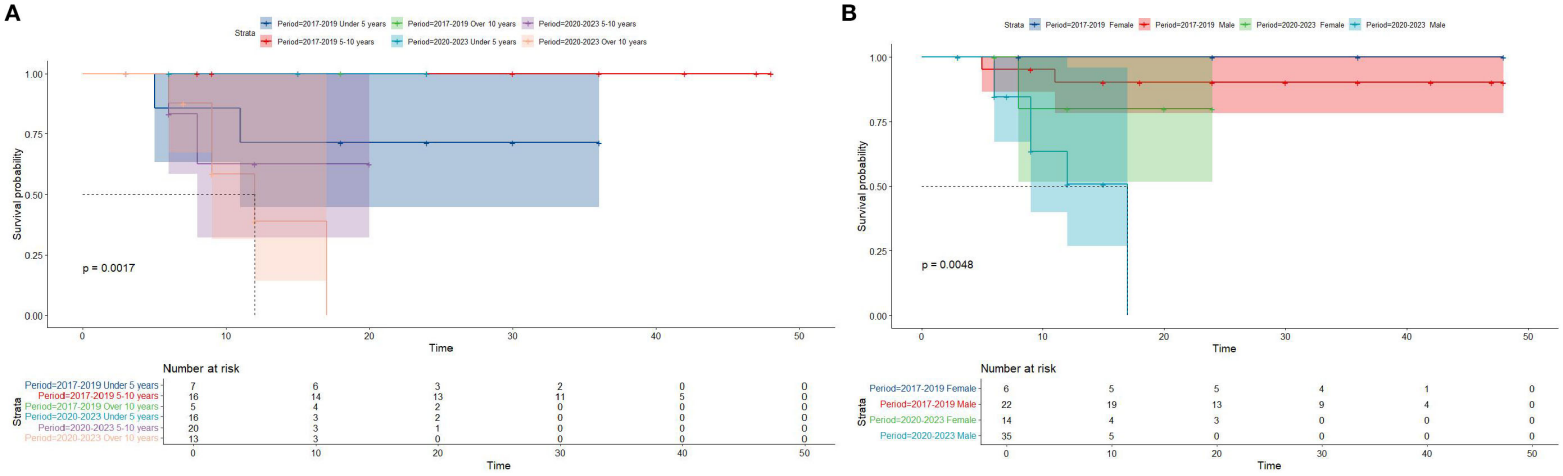
Overall survival in participants with Lymphoblastic Lymphoma from the Chinese cohort by period year in different age and gender groups (age group: **(A)**; gender group: **(B)**).

**Figure 4:**
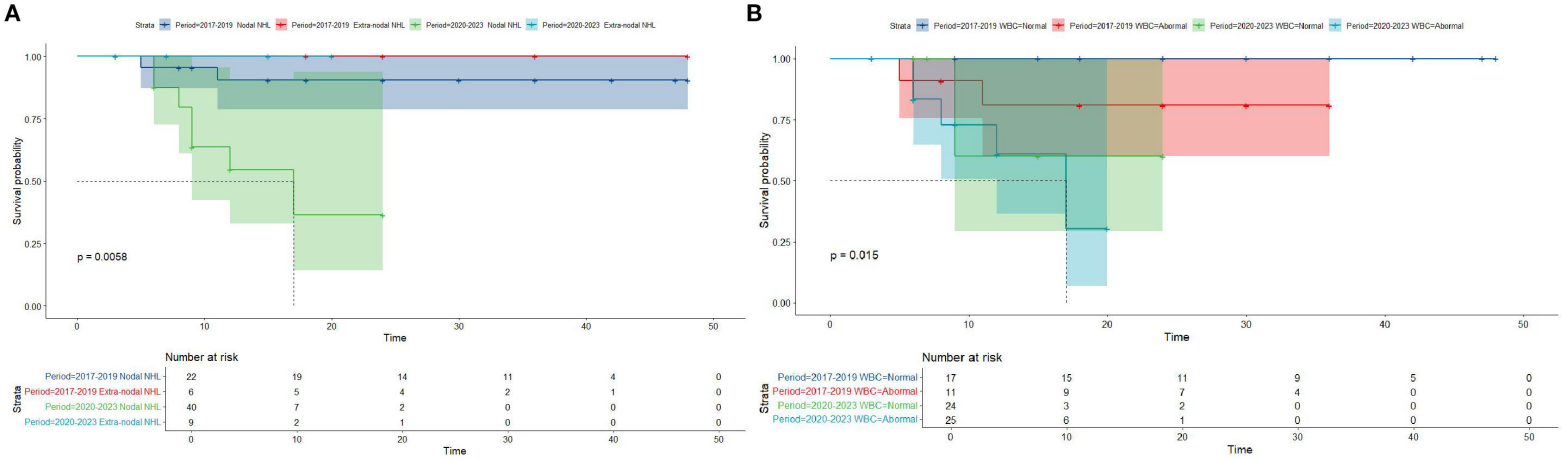
Overall survival in participants with Lymphoblastic Lymphoma from the Chinese cohort by period year in different primary site and WBC groups (age group: **(A)**; gender group: **(B)**).

**Figure 5:**
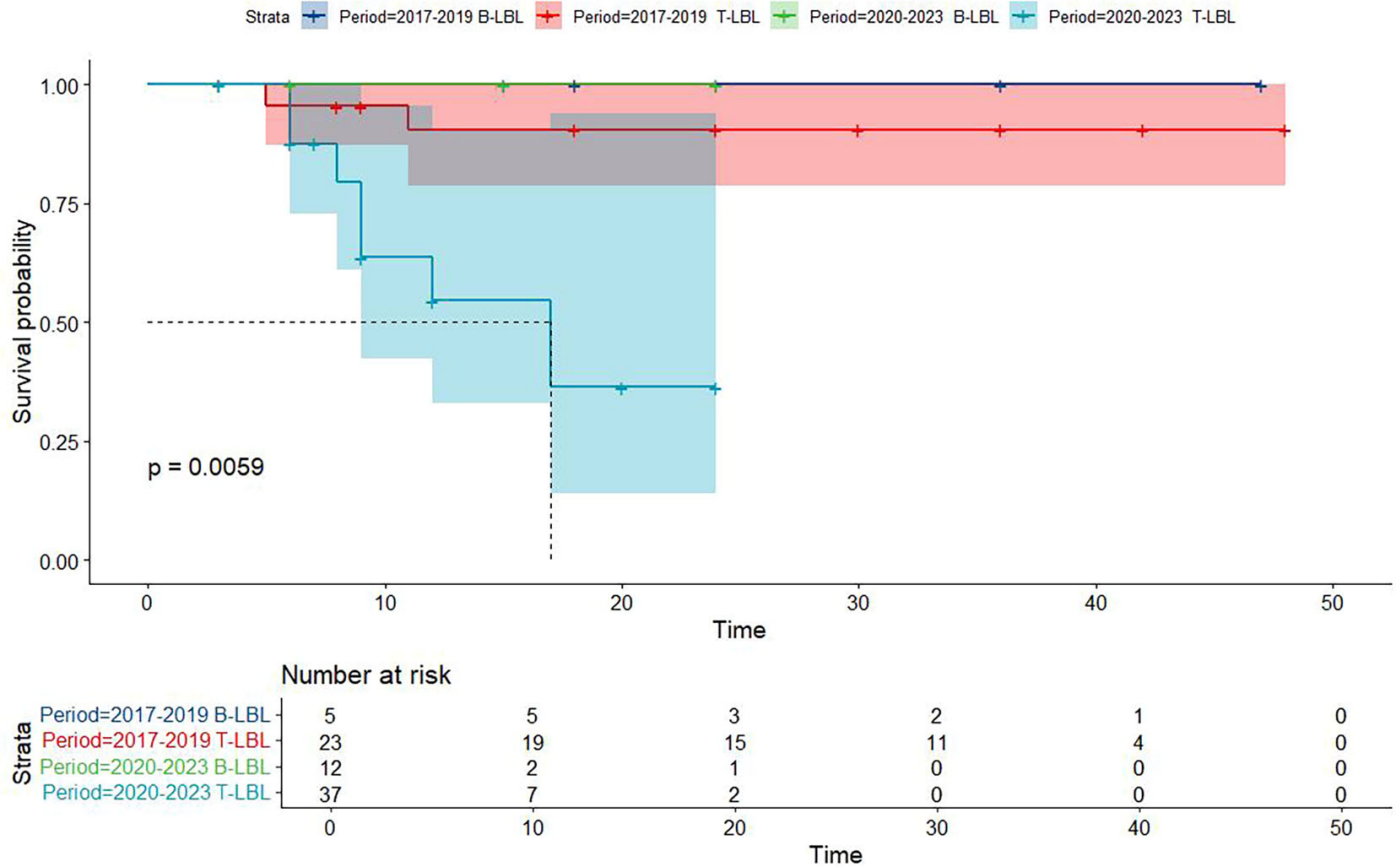
Overall survival in participants with Lymphoblastic Lymphoma from the Chinese cohort by period year in different histology type groups.

**Figure 6:**
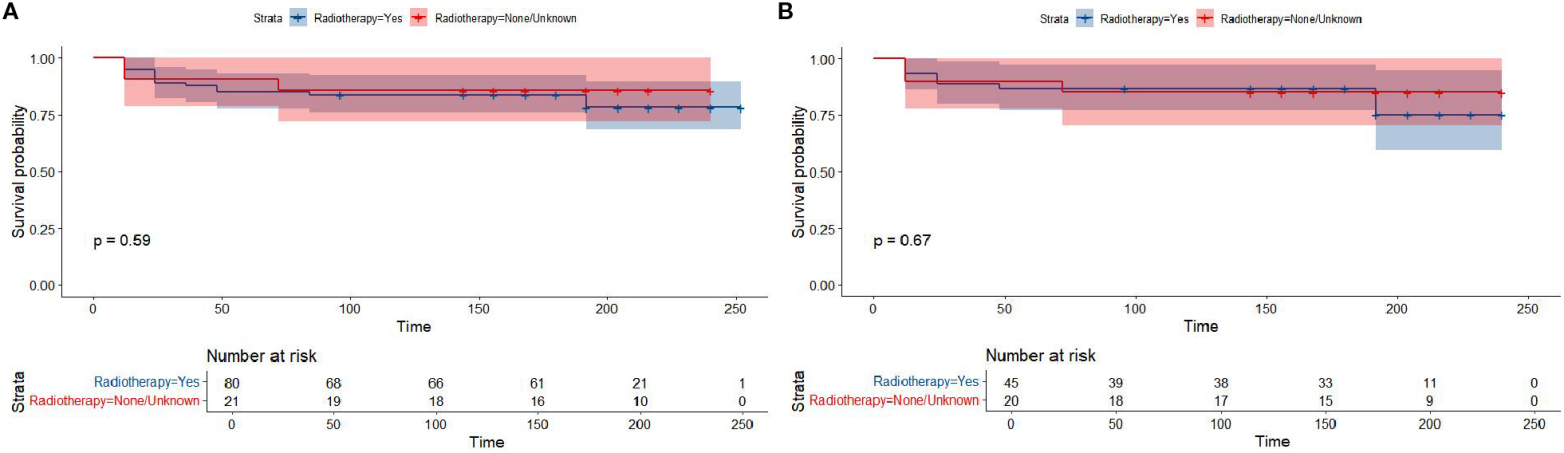
Overall survival in participants with Lymphoblastic Lymphoma from the SEER cohort by radiotherapy or un-radiotherapy groups (All-LBL: **(A)**; T-LBL: **(B)**).

Kaplan-Meier survival analysis of patients from different histology groups revealed no significant differences in overall survival between B-LBL and T-LBL patients in either cohort (SEER: P = 0.79, China: P = 0.14; **Figure 1**). In the Chinese cohort, patients diagnosed with LBL and T-LBL in the 2017–2019 period had relatively better prognosis compared to those diagnosed in the 2020–2023 period (**Figure 2**). Subgroup analysis demonstrated significant differences in overall survival between LBL patients from the 2017–2019 and 2020–2023 periods, based on age, gender, white blood cell count (WBC), primary site, and histological type (**Figures 3**–**5**). Additionally, in the SEER cohort, whether patients underwent radiotherapy did not have a significant impact on the overall survival of LBL and T-LBL patients (**Figures 6**).

## 4 Discussion

In this study, we conducted a comparative prognostic analysis of pediatric lymphoblastic lymphoma (LBL) using data from two distinct cohorts: the China-LBL cohort and the United States SEER-LBL cohort. Our findings highlight several important clinical characteristics and survival outcomes, shedding light on regional differences, treatment practices, and the potential prognostic implications of certain variables.

### 4.1 Prognostic factors in the Chinese and SEER cohorts

Our analysis revealed that pediatric LBL patients in the Chinese cohort treated between 2017 and 2019 had significantly better overall survival compared to those treated between 2020 and 2023. This discrepancy in survival outcomes is noteworthy, especially considering that both periods employed similar treatment regimens, such as the improved BCH-2017-LBL protocols. The reasons for this difference remain speculative, but several potential factors could explain the observed trend.

One possible explanation is the impact of the COVID-19 pandemic, which began in 2020 and disrupted healthcare systems worldwide. During the pandemic, many hospitals faced challenges such as resource constraints, delays in diagnosis, treatment interruptions, and restrictions on in-person visits, all of which may have negatively influenced patient care and outcomes (21–23). The Chinese cohort treated between 2020 and 2023 may have experienced such disruptions, leading to delays in treatment initiation, reduced frequency of monitoring, and interruptions in chemotherapy cycles or supportive care. In contrast, the period from 2017 to 2019, prior to the pandemic, likely benefited from a more stable healthcare environment, with fewer treatment delays and greater consistency in patient management.

Additionally, the psychological and physical strain caused by the pandemic, such as heightened anxiety, reduced adherence to treatment, and limited access to healthcare facilities, may have further compounded the challenges faced by patients and caregivers during the 2020–2023 period. These factors could contribute to the relatively poorer survival outcomes seen in this later period, despite the use of established treatment protocols. However, the reasons behind the improved survival between 2017 and 2019 remain speculative, but may include factors such as better patient selection, fewer treatment interruptions, or improvements in supportive care, such as infection control or management of chemotherapy-induced toxicity.

The favorable outcomes observed in the earlier period could also be linked to the inherent biological differences between patient cohorts, such as genetic predispositions, disease biology, or variations in disease stage at diagnosis. It is worth noting that the SEER cohort did not show the period-related survival differences, which may be reflective of differences in treatment protocols or healthcare infrastructure between the U.S. and China.

### 4.2 Impact of treatment and staging differences

Notably, the China-LBL cohort had a significantly lower proportion of patients with stage I disease, with a majority of cases diagnosed at stage III or IV, in contrast to the SEER cohort, where stage I patients were more common. This difference in staging could potentially explain some of the survival discrepancies observed between the two cohorts. Stage at diagnosis is one of the most important prognostic factors in LBL, and it is well-established that patients with advanced-stage disease tend to have poorer outcomes despite aggressive treatment (16, 17).

The two cohort also displayed a marked difference in the use of radiotherapy. While no patients in the Chinese cohort received radiotherapy, 20.8% of the SEER cohort received radiotherapy as part of their treatment regimen. The absence of radiotherapy in the Chinese cohort could be attributed to the adoption of modified chemotherapy protocols that emphasize less invasive interventions, such as the avoidance of cranial radiation therapy (CRT) in high-risk patients. This approach is supported by several studies that have demonstrated the potential for chemotherapy alone to provide comparable survival outcomes, while also minimizing long-term neurological sequelae associated with CRT (24, 25). In addition, a marked difference in the use of radiotherapy (RT) was observed between the two cohorts, which primarily reflects the evolution of treatment paradigms over time. In the SEER-LBL cohort (2000-2009), 20.8% (21/101) of patients received RT. This is consistent with the historical standard of care during that era, where consolidative RT was more commonly employed for patients with high-risk features, such as bulky mediastinal disease or central nervous system involvement (24, 26). In contrast, no patients in the contemporary China-LBL cohort (2017-2023) received RT. This shift in practice is driven by accumulating evidence demonstrating that modern, intensified chemotherapy protocols alone can achieve comparable survival outcomes while successfully avoiding the long-term sequelae associated with RT, such as secondary malignancies and neurocognitive deficits (27, 28). The absence of RT in our China cohort underscores the successful adoption of this refined therapeutic strategy. Interestingly, our analysis found no significant difference in overall survival between patients who received radiotherapy and those who did not in the SEER cohort, suggesting that modern chemotherapy regimens may offer sufficient efficacy in the absence of radiotherapy, particularly in the context of T-LBL.

### 4.3 Histological subtypes and survival outcomes

Despite differences in treatment protocols and patient characteristics, no significant survival difference was observed between T-LBL and B-LBL patients in either the SEER or China cohort. This finding is consistent with several prior studies that have suggested that, although T-LBL and B-LBL may have distinct biological features, their prognostic significance may not always translate into differences in overall survival when treated with standard ALL-based regimens (29, 30). Our results support the notion that both T-LBL and B-LBL are effectively managed using risk-adapted chemotherapy strategies, with survival outcomes largely dependent on factors such as disease stage, treatment adherence, and access to supportive care.

### 4.4 Subgroup analysis: prognostic factors in the Chinese cohort

Subgroup analyses within the Chinese cohort further identified significant differences in overall survival based on age, gender, white blood cell count (WBC), histological subtype, and primary site. These findings reinforce the importance of individualized risk stratification in the management of pediatric LBL. Age is particularly noteworthy, as younger patients tend to have better outcomes (31), likely due to a higher likelihood of achieving complete remission and better tolerance of aggressive chemotherapy regimens.

The analysis also suggests that specific clinicopathological factors, such as high WBC counts and certain histological subtypes, may serve as indicators of worse prognosis, aligning with previous studies on T-ALL and LBL. For instance, elevated WBC counts at diagnosis have been consistently associated with poorer survival outcomes (32, 33), likely due to the increased tumor burden and more aggressive disease biology. Similarly, the presence of certain genetic or molecular markers could help refine risk stratification models in future studies.

## 5 Limitations and future directions

Despite the valuable insights provided by this study, there are several limitations that should be considered. First, the retrospective design of the study may introduce biases related to patient selection and data collection. A limitation specific to the China-LBL cohort is the uneven distribution of patients across the two compared time periods, with a majority (49 [63.6%]) diagnosed between 2020 and 2023 compared to those diagnosed between 2017 and 2019 (28 [36.4%]). This imbalance may have influenced the comparative survival analysis. As it may be attributable to factors beyond therapeutic efficacy alone, most notably the disruptive impact of the COVID-19 pandemic on healthcare systems and the inherently shorter follow-up duration for later patients, which more fully captures early adverse events. Furthermore, the relatively short follow-up period for the Chinese cohort (median of 8 months) limits our ability to fully assess long-term survival outcomes and late-onset complications, such as secondary malignancies or cognitive deficits following treatment. In contrast, the SEER cohort benefited from a longer median follow-up (180 months), which allowed for a more comprehensive assessment of survival patterns over time.

Additionally, the lack of detailed molecular data in both cohorts restricts our ability to analyze the role of specific genetic mutations or alterations in prognosis. Future prospective studies, ideally multicenter and multinational, are needed to explore these molecular factors in greater depth and to evaluate the long-term impact of treatment strategies, such as the omission of radiotherapy, on survival and quality of life.

## 6 Conclusion

In conclusion, this study provides a comparative analysis of pediatric LBL from two distinct cohorts, highlighting the prognostic impact of treatment period, stage at diagnosis, and regional treatment practices. While survival outcomes between T-LBL and B-LBL were not significantly different in either cohort, important differences in clinical features and treatment regimens were observed. The results underscore the importance of early diagnosis, risk stratification, and the potential for evolving treatment strategies to improve survival outcomes in pediatric LBL. Further studies are warranted to explore the molecular underpinnings of this disease and refine treatment approaches to minimize long-term sequelae while maximizing survival.

## Data Availability

The raw data supporting the conclusions of this article will be made available by the authors, without undue reservation.
